# Supplementary material for: Time series experimental design under one-shot sampling: The importance of condition diversity
Source: PLoS One. 2019 Oct 31;14(10):e0224577. doi: 10.1371/journal.pone.0224577 (PMC6822768; doi:10.1371/journal.pone.0224577)
Supplement: S1 Appendix — The parameters A, γ, σ, and σte are assumed unknown and jointly estimated in GLRT. (PDF) [file pone.0224577.s001.pdf]

Supplementary information

Appendix

S1 Joint estimation for single-gene autoregulation recovery

The simulation results in Fig 4 are for the GLRT estimator that is assumed to know the level of the driving noise random variables (i.e. the $W_j^k(t)$ random variables), which for brevity we normalized to one. To account for not knowing the variance we could multiply the W's by a positive parameter σ, such that σ that is not known to the algorithm. In addition, it was assumed that the GLRT knows the variance of the observation noise, σ_{te}^2. This section presents additional simulations, for a GLRT estimator that needs to estimate all four parameters, A, γ, σ, and σ_{te}, again for recovery of the sign of regulation for a single gene. The sign error rates with the true $A = 0.1, 0.5$ and one-shot and multi-shot sampling are shown in Fig 1S. It can be seen in comparison with Fig 4 that the performance of GLRT is not significantly worse without the knowledge of σ and σ_{te}.

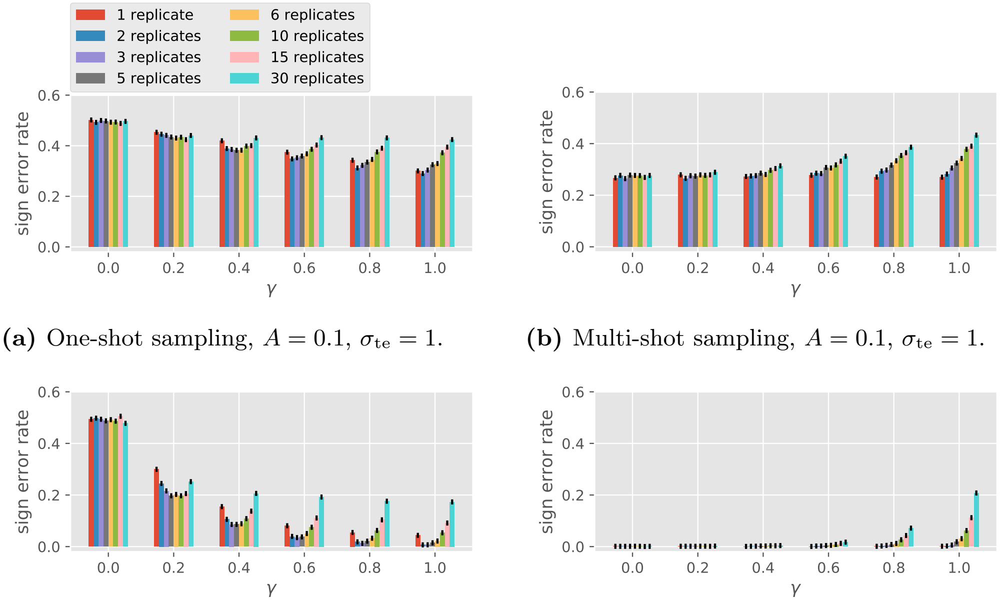

(a) One-shot sampling, $A = 0.1$, $\sigma_{\mathrm{te}} = 1$.

(b) Multi-shot sampling, $A = 0.1$, $\sigma_{\mathrm{te}} = 1$.

(c) One-shot sampling, $A = 0.5$, $\sigma_{\mathrm{te}} = 1$.

(d) Multi-shot sampling, $A = 0.5$, $\sigma_{\mathrm{te}} = 1$.

Fig 1S. Error rates of the GLRT for the sign of a single gene with confidence intervals based on 10 000 simulations. All four parameters (A, γ, σ and σ_{te}) are unknown to the algorithm.

The scatter plots of the estimate $\hat{A}$ against the actual A and that of the estimate $\hat{\gamma}$ against the actual γ with different joint prior on A and γ and $C = 10$, $R = 3$, $T = 6$ are shown in Figs 2S–6S. Each plot is based on 1000 simulations. For all these figures, the

plots on the left are for one-shot sampling and the plots on the right are for multi-shot sampling. Also, for all these figures the GLRT algorithm did not have access to σ and σ_{te}. Fig 2S shows scatterplots that indicate how well A and γ can be estimated when the true value of (A, γ) is uniformly distributed over $[-1, 1] \times [0, 1]$. Figs 3S–6S show how well A and γ can be estimated when only one of these parameters is uniformly distributed and the other is fixed at 0.1 or 0.5.

We can see that for all cases shown, the estimates of A and γ are more accurate for multi-shot sampling vs. one-shot sampling. Comparing Figs 3S and 4S, we see that increasing A slightly helps the estimation of γ. Comparing Figs 5S and 6S, we see that increasing γ greatly improves the estimation of A for one-shot sampling, while it slightly hurts the estimation of A for multi-shot sampling. These observations are consistent with the performance of GLRT for sign recovery seen in Figs 4 and 1S. Namely, for one-shot sampling the performance significantly improves as γ increases, while for multi-shot sampling the performance slightly degrades as γ increases (but it is much worse for $\gamma \approx 1$).

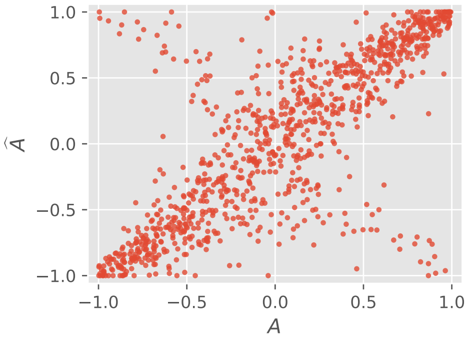

(a) $\hat{A}$ against A with one-shot sampling. $\rho = 0.76$.

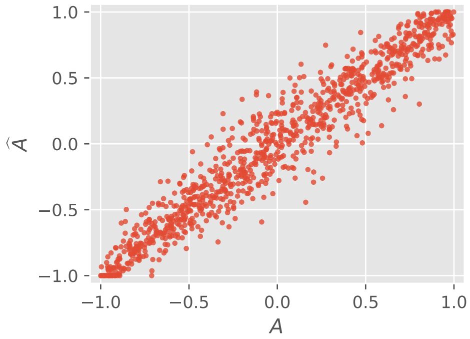

(b) $\hat{A}$ against A with multi-shot sampling. $\rho = 0.96$.

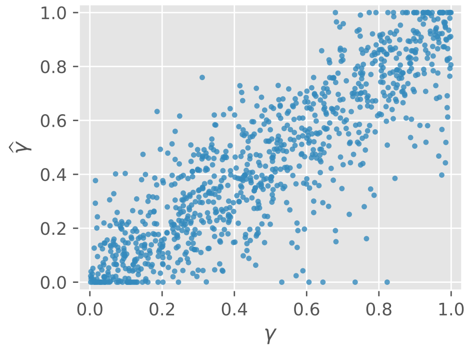

(c) $\hat{\gamma}$ against γ with one-shot sampling. $\rho = 0.87$.

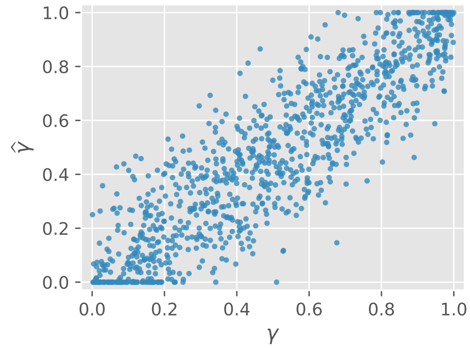

(d) $\hat{\gamma}$ against γ with multi-shot sampling. $\rho = 0.89$.

Fig 2S. Joint estimation scatter plots with $\sigma = 1$, $\sigma_{\mathrm{te}} = 1.0$ and $(A, \gamma) \sim \mathsf{Unif}([-1, 1] \times [0, 1])$.

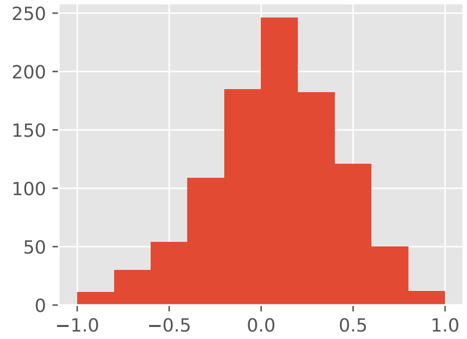

(a) Histogram of $\hat{A}$ for fixed $A = 0.1$ with one-shot sampling.

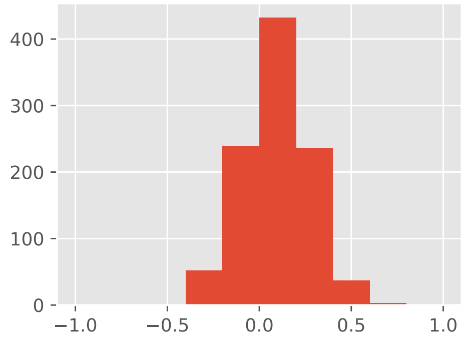

(b) Histogram of $\hat{A}$ for fixed $A = 0.1$ with multi-shot sampling.

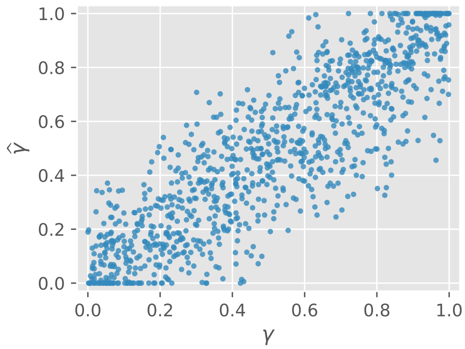

(c) $\hat{\gamma}$ against γ with one-shot sampling and fixed $A = 0.1$. $\rho = 0.87$.

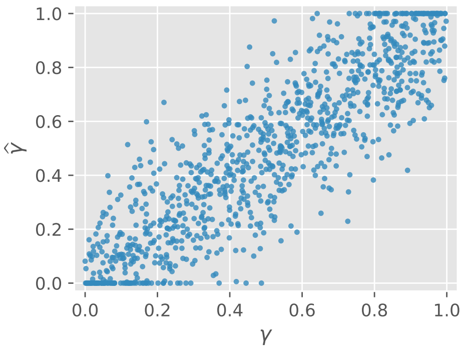

(d) $\hat{\gamma}$ against γ with multi-shot sampling and fixed $A = 0.1$. $\rho = 0.88$.

Fig 3S. Joint estimation plots with $\sigma = 1$, $\sigma_{\mathrm{te}} = 1$, fixed $A = 0.1$ and $\gamma \sim \mathsf{Unif}([0, 1])$.

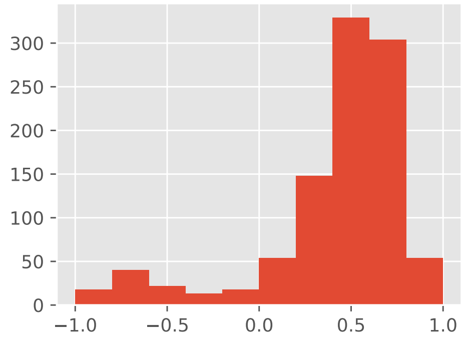

(a) Histogram of $\hat{A}$ for fixed $A = 0.5$ with one-shot sampling.

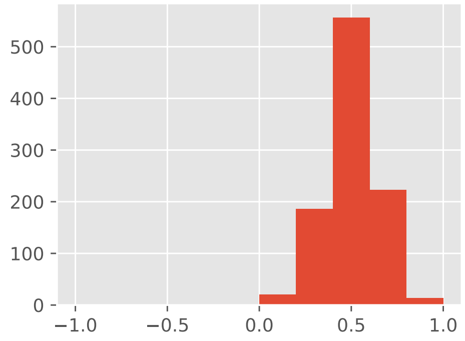

(b) Histogram of $\hat{A}$ for fixed $A = 0.5$ with multi-shot sampling.

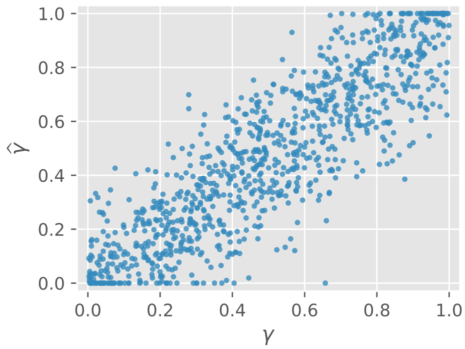

(c) $\hat{\gamma}$ against γ with one-shot sampling and fixed $A = 0.5$. $\rho = 0.88$.

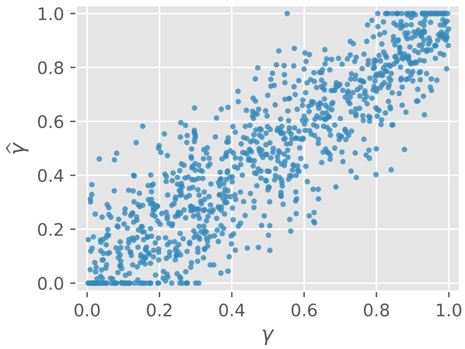

(d) $\hat{\gamma}$ against γ with multi-shot sampling and fixed $A = 0.5$. $\rho = 0.89$.

Fig 4S. Joint estimation plots with $\sigma = 1$, $\sigma_{\text{te}} = 1$, fixed $A = 0.5$ and $\gamma \sim \mathsf{Unif}([0, 1])$.

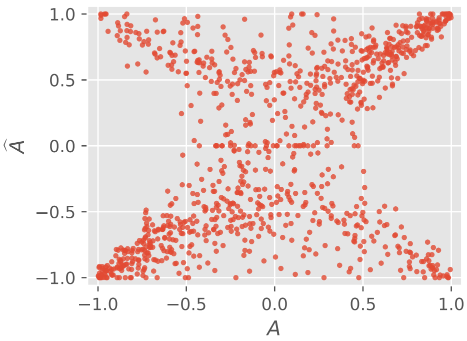

(a) $\hat{A}$ against A with one-shot sampling and fixed $\gamma = 0.1$. $\rho = 0.41$.

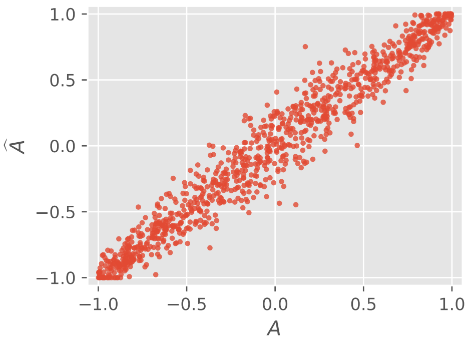

(b) $\hat{A}$ against A with multi-shot sampling and fixed $\gamma = 0.1$. $\rho = 0.98$.

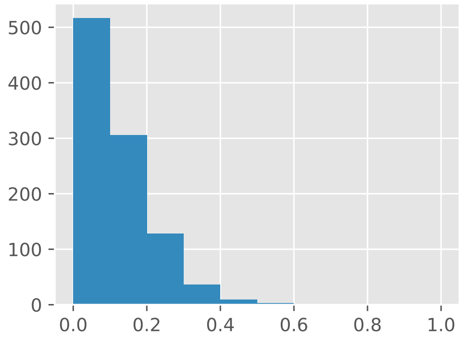

(c) Histogram of $\hat{\gamma}$ for fixed $\gamma = 0.1$ with one-shot sampling.

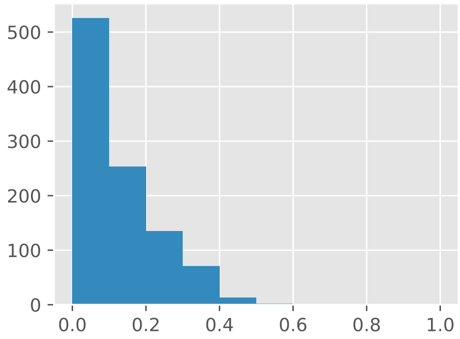

(d) Histogram of $\hat{\gamma}$ for fixed $\gamma = 0.1$ with multi-shot sampling.

Fig 5S. Joint estimation scatter plots with $\sigma = 1$, $\sigma_{\text{te}} = 1$, fixed $\gamma = 0.1$ and $A \sim \mathsf{Unif}([-1, 1])$.

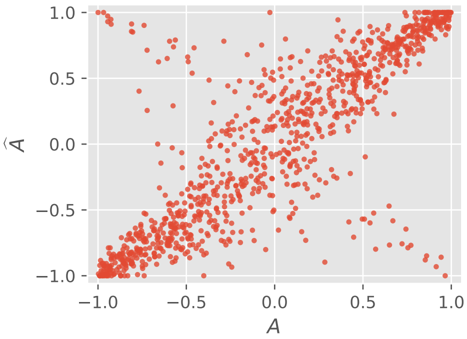

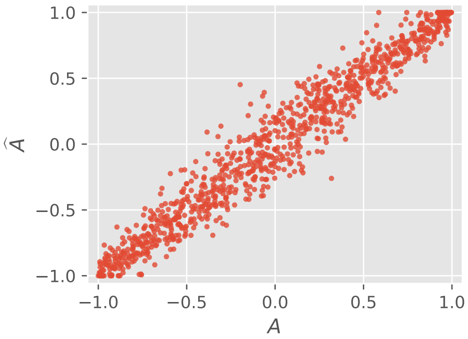

(a) $\hat{A}$ against A with one-shot sampling and fixed $\gamma = 0.5$. $\rho = 0.82$.

(b) $\hat{A}$ against A with multi-shot sampling and fixed $\gamma = 0.5$. $\rho = 0.97$

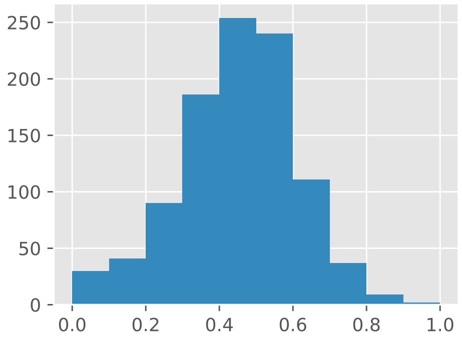

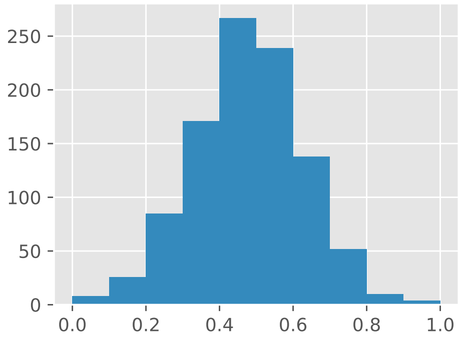

(c) Histogram of $\hat{\gamma}$ for fixed $\gamma = 0.5$ with one-shot sampling.

(d) Histogram of $\hat{\gamma}$ for fixed $\gamma = 0.5$ with multi-shot sampling.

Fig 6S. Joint estimation plots with $\sigma = 1$, $\sigma_{\text{te}} = 1$, fixed $\gamma = 0.5$ and $A \sim \mathsf{Unif}([-1, 1])$.
