# Supplementary material for: Time series experimental design under one-shot sampling: The importance of condition diversity
Source: PLoS One. 2019 Oct 31;14(10):e0224577. doi: 10.1371/journal.pone.0224577 (PMC6822768; doi:10.1371/journal.pone.0224577)
Supplement: S2 Appendix — The random network prior distribution used to generate the multi-gene network. (PDF) [file pone.0224577.s002.pdf]

Supplementary information

Appendix

S2 Split Gaussian network prior

Let the prior of the adjacency matrix be such that the in-degree of each vertex is uniform over $\{0, 1, \ldots, d_{\max}\}$ for some $d_{\max} \geq 1$, and the set of parents is selected uniformly at random from the rest of vertices given the in-degree. For each edge from vertex i to vertex j, let the corresponding element in the adjacency matrix be split Gaussian distributed with unit gap and unit variance (i.e., $a_{ij} = (0.5 + (0.554553)|Z|)\,\mathrm{sgn}(Z)$ for $Z \sim \mathcal{N}(0, 1)$.). The degradation and the autoregulation are ignored; i.e., $a_{ii} = 0$ for all i. We call this prior the *split Gaussian prior* with max in-degree $d_{\max}$.
