# Supplementary material for: Time series experimental design under one-shot sampling: The importance of condition diversity
Source: PLoS One. 2019 Oct 31;14(10):e0224577. doi: 10.1371/journal.pone.0224577 (PMC6822768; doi:10.1371/journal.pone.0224577)
Supplement: Attachment — Submitted filename: response.pdf [file pone.0224577.s003.pdf]

October 3, 2019

Dear Dr. Abel:

I am pleased to submit a revision of our original research article entitled "Time series experimental design under one-shot sampling: The importance of condition diversity" by Xiaohan Kang, Bruce Hajek, Faqiang Wu, and Yoshie Hanzawa for consideration for publication in PLOS ONE. Please find our responses to the reviewer's and the editor's comments below.

Reviewer #1

Summary: *This papers explores the effect of condition variance and biological variance under one-shot and multi-shot sampling. It provides a general model for network representation when these parameters are known. Further, the authors describe the relationship between the sampling regimes and their representation in the model described. Finally, the authors simulation from this model and describe the performance of two-estimators under different configurations.*

Response: We thank the reviewer for the careful reading of the manuscript.

Comments: *Things I would like to see:*

1. *A table describing the simulations performed and summarizing the behavior.*

   Response: We now summarize the simulations in Table 1 on page 20.

2. *A description of how the adjacency matrix A was chosen from simulations.*

   Response: We now emphasize on page 17 that the adjacency matrix A is chosen i.i.d. from the prior distribution described in Appendix S2.

   > So instead of fixing a ground truth A as in Fig 4, we fix a prior distribution of A with split Gaussian prior described in Appendix S2 (note we assume the knowledge of no autoregulation), and choose A i.i.d. from the prior distribution with $d_{\max} = 3$.

3. *An application on "real" data.*

   Response: We could not find a real biological dataset with both one-shot and multi-shot samples and a reliable ground truth network. As we discuss on pages 22–25, most expression datasets are one-shot data, and there is no well-accepted ground

truth. Thus, to address the reviewer's comment, we generate expression data from a most-accepted *Arabidopsis* circadian clock model using a DREAM-challenge-like SDE model and run BSLR on both the one-shot and the multi-shot datasets. We show that even for such a sophisticated model with nonlinear regulation, continuous-time dynamics, unobserved cytoplasmic and nuclear protein concentrations, and very limited samples, the simple BSLR algorithm demonstrates a clear improvement from one-shot sampling to multi-shot sampling. The results also show that one-shot data is better paired with replicate averaging, while multi-shot data is better paired with no replicate averaging, illustrating the effect of sampling method on the data analysis decisions.

See the new discussion section "A case study on *Arabidopsis* circadian clock network" on pages 25–28 for the details.

4. *A comparison of performance to other methods on some of the DREAM data.*

   Response: Because the focus of the paper is on the difference between one-shot and multi-shot sampling methods and how the difference can be mitigated by treating one-shot data under the same conditions as multi-shot data, we feel that comparison to algorithms that do not take this difference into considerations would be a digression. As a result, we choose GLRT (for single-gene recovery) and BSLR (for multi-gene recovery), which are theoretically optimal, practically competitive, and intuitively understandable algorithms, as opposed to other variants of approximations. We believe the results on the two simple algorithms are easier to interpret for the purpose of this paper.

   As for the DREAM challenge data, neither the real biological data nor the *in silico* data includes one-shot sampling. Hence they are not suitable for validation of the results in this paper. However, as we mentioned in the response of the previous comment, we do include the simulated expression data using SDE model similar to that of the *in silico* data in the DREAM challenges for a most-accepted *Arabidopsis* circadian clock network.

5. *The section "On biological replicates" is a bit odd and seems tacked on. Particularly, the discussion of differential expression tools seems odd. The goal in those tools is not to infer gene regulation, but to infer whether the expression of some set of genes is changed given some experimental perturbation. Not sure what you mean here (line 551):*

"It is then not clear whether replicates bring more benefit than the sheer additional amount of data compared to samples under different conditions."

Response: We rewrite the discussion section "On biological replicates" to tie in with the one-shot and multi-shot sampling analysis. See details on pages 24, 25. We also clarify the sentence on line 551 by replacing it with the following.

> It is then not clear whether it is better to have more replicates under the same condition, or to have more conditions, for a fixed total number of samples.

Editor's comment:

In the reviewer comments below, I would emphasize the exploration of actual biological data and the comparison of performance to one or two other methods.

Response: See our responses to comments 3 and 4 by Reviewer #1.

Sincerely,

Xiaohan Kang, PhD

Postdoc, Coordinated Science

Laboratory and Department of

Electrical and Computer Engineering

University of Illinois at

Urbana–Champaign
